# Treatment of open ankle dislocation without associated fractures in a young athlete using external fixation and ligament repair with suture tape augmentation

**DOI:** 10.1186/s12891-020-03378-z

**Published:** 2020-06-05

**Authors:** Ryosuke Kawai, Itaru Kawashima, Makoto Tsukada, Takashi Tsukahara, Hideyuki Aoshiba

**Affiliations:** Department of Orthopedic Surgery, Asahi University Hospital, 3-23 Hashimoto town, Gifu, 500-8523 Japan

**Keywords:** Pure ankle dislocation, Tibiotalar, Young athlete, Anterior talofibular ligament, Suture tape augmentation

## Abstract

**Background:**

Ankle dislocation without fracture is an extremely rare injury because it is usually accompanied by concomitant malleolar fractures from the anatomical and mechanical viewpoints.

**Case presentation:**

We report the case of a 17-year-old woman who was injured while playing basketball. Her ankle was swollen and deformed. Plain X-ray revealed tibiotalar dislocation in the medial direction without any fractures.

Immediate reduction and ligament repair using suture tape augmentation were performed.

**Conclusions:**

At 5 months postoperatively, the patient returned to playing basketball without any complaints. After an additional 2 months, the patient participated and played in the Winter Cup 2019 (the national high school basketball tournament in Japan) at the previous performance level.

## Background

The occurrence of ankle dislocation without accompanying fractures is an extremely rare event [1–4]. This condition is referred to as “pure dislocation” because it is usually accompanied by concomitant malleolar fractures from an anatomical and mechanical perspective. Moreover, the gold standard treatment for this condition has not yet been established.

In the present report, we describe a case of ankle dislocation that occurred without associated fractures in a young athlete. The patient was an elite basketball player in high school and had injured her right ankle during a contact play in basketball. To facilitate early return to basketball, external fixator and ligament repair with suture tape augmentation to repair the damaged anterior talofibular ligament (ATFL), calcaneofibular ligament **(**CFL), and deltoid ligament (DL).

The patient was able to return to playing basketball at the previous performance level 5 months postoperatively. The Japanese Society for Surgery of the Foot (JSSF) ankle/hindfoot scale score [5, 6] was excellent (100/100), and 2 months subsequently, the patient participated and played in the Winter Cup 2019 (the national high school basketball tournament in Japan).

## Case presentation

**Fig. 1):**
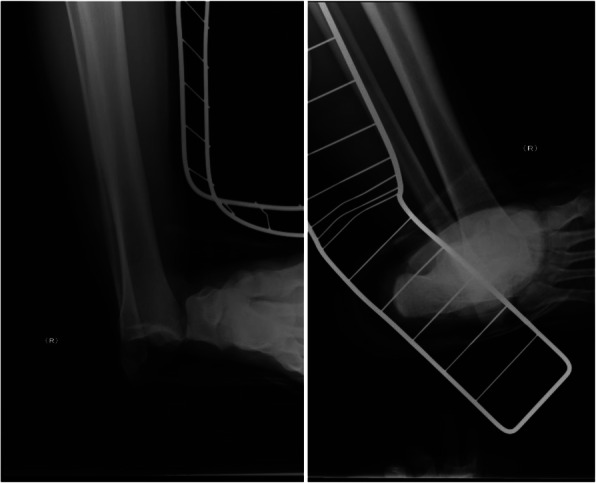
Plain x-ray image obtained at the first visit to our hospital. The tibiotalar joint was found to be dislocated in the medial direction without any fractures

A 17-year-old woman fell and injured her right ankle during contact play in basketball. She was immediately transported to our hospital, where her ankle was found to be swollen and deformed. A 4.5-cm transverse wound was noted at the distal fibula. Plain X-ray revealed tibiotalar dislocation in the medial direction without any fractures (Fig. 1).

## Surgical technique

### Reduction using external fixation

**Fig. 2:**
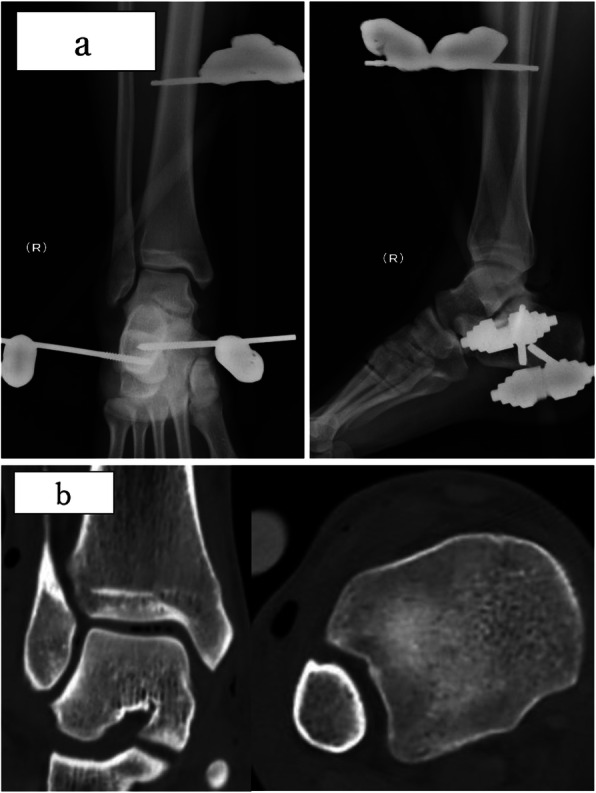
**a** Plain x-ray image obtained after reduction was performed using external fixation. Reduction was excellent and the ankle was stabilized in a neutral position (ankle 0° position). **b** CT performed after reduction revealed no malleolar fractures and normal tibia-fibula distance

Emergency washout and debridement were performed with primary closure of the wound, and reduction using an external fixator under lumbar anesthesia was performed on the night of the injury. One Schanz screw was inserted into the distal tibia and two into the calcaneus; the ankle was stabilized at the neutral position (ankle 0°position) (Fig. 2a). The postoperative computerized tomography (CT) also revealed no malleolar fractures and syndesmosis injury. (Fig. 2b).

### Repair of the ruptured ATFL and CFL

After 2 weeks of external fixation, the severe swelling gradually improved and the condition of the soft tissue and wound was good. The operation was performed under lumbar anesthesia. First, the external fixator was completely removed; the varus and valgus stress views were confirmed using fluoroscopy. Subsequently, medial and lateral instabilities of the ankle joint were revealed, and ligament repair was performed.

A 4-cm oblique skin incision was made 1 cm distal to the fibula tip. The ATFL and CFL were found to be completely ruptured near the proximal attachment. Primary repair of the ruptured ATFL and CFL was performed using the DX FiberTak® all-soft suture anchor (Arthrex Inc., Naples, FL). A DX FiberTak® all-soft suture anchor was inserted between the ATFL and CFL at the anatomical origin of the distal fibula; both ligaments were subsequently lifted up to the proximal attachment and repaired using sutures.

**Fig. 3:**
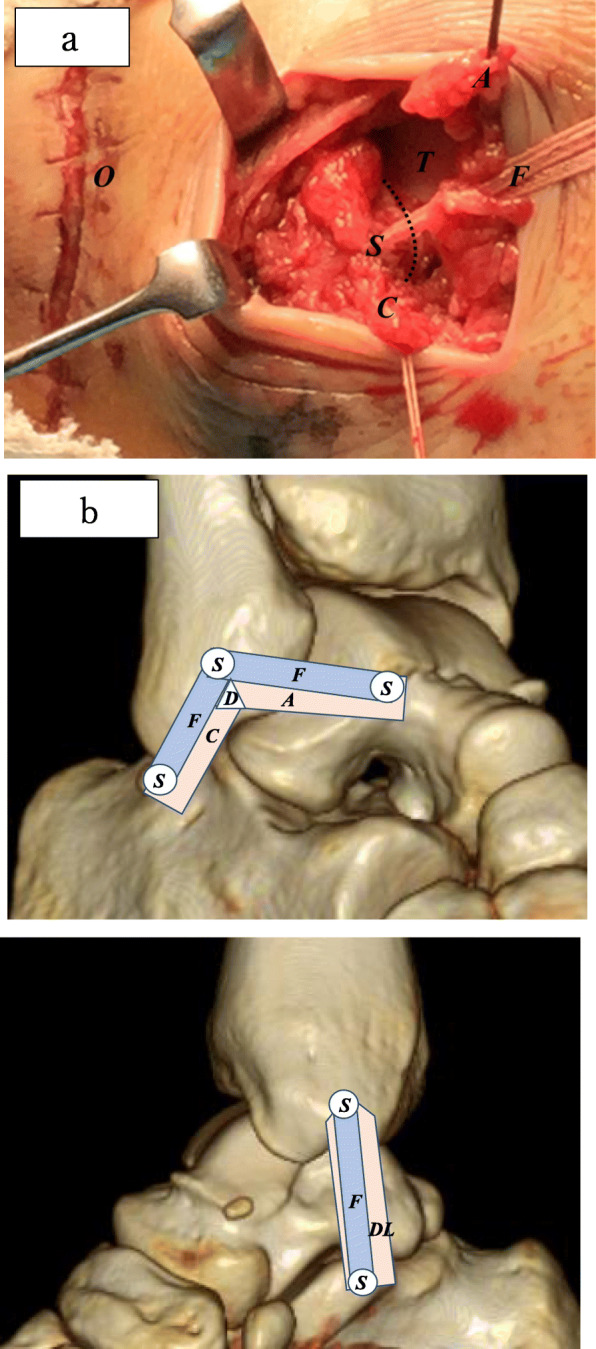
**a** Intraoperative photograph of anterior talofibular ligament/calcaneofibular ligament repair and suture tape augmentation. The anterior talofibular ligament (ATFL) and calcaneofibular ligament (CFL) were completely ruptured near the proximal attachment. A BioComposite SwiveLock® suture anchor with a FiberTape® was placed just proximal between the ATFL and CFL anatomical origin of the distal fibula. Key: A, anterior talofibular ligament; C, calcaneofibular ligament; F, FiberTape®; O, open wound at injury; T, talus; S, BioComposite SwiveLock® suture anchor; dotted curved line, leading edge of the distal fibula. **b** Schematic of suture tape augmentation for the lateral sides and the medial sides of the ankle joint. FiberTape® in the anterolateral distal fibula and talus/calcaneus along the lateral articular margin is secured with the BioComposite SwiveLock®⎕ anchors at the origin and insertion points of the ATFL/CFL. FiberTape® is secured with the BioComposite SwiveLock®⎕ anchors at the origin and insertion points of the DL. Key: A, anterior talofibular ligament; C, calcaneofibular ligament; D, DX FiberTak® all-soft suture anchor; F, FiberTape®; S, BioComposite SwiveLock® suture anchor; DL, deltoid ligament

Next, suture tape augmentation was performed on the repaired ATFL and CFL. We placed a proximal 4.75-mm BioComposite SwiveLock® suture anchor with FiberTape® (Arthrex Inc., Naples, FL; 2.0-mm-wide suture tape composed of braided ultra-high-molecular-weight polyethylene and polyester) just proximal of the DX FiberTak® anchor. While maintaining adequate tension of the FiberTape®, a 3.5-mm BioComposite SwiveLock®⎕ anchor was placed to secure the FiberTape® each into the ATFL attachment on the talar neck and the CFL attachment on the lateral wall of the calcaneal body in the neutral ankle position with a bump placed under the tibia to avoid any anterior translation (Figs. 3a, b).

### Repair of the damaged DL

The superficial and deep DLs were found to be damaged in the mid portion. A 3-cm oblique skin incision was made just distal of the medial malleolar, and primary suture repair was performed using 0 ETHIBOND.

Suture tape augmentation was subsequently performed. The 4.75-mm BioComposite SwiveLock® anchor with FiberTape® was placed in the medial malleolus at the center of the attachment of the DL. Another 3.5-mm BioComposite SwiveLock® anchor to secure the FiberTape® was placed in the distal sustentaculum tali of the calcaneus under fluoroscopy while confirming the adequate tension of the FiberTape® in the neutral ankle position.

### Postoperative rehabilitation

After 2 weeks of ankle casting, range-of-motion exercises for the ankle were initiated and full weight bearing was allowed. At 3 months postoperatively, jogging was started, and at 4 months postoperatively, the patient was allowed to return to basketball practice and participate in basic training such as dribbling and shooting.

**Fig. 4):**
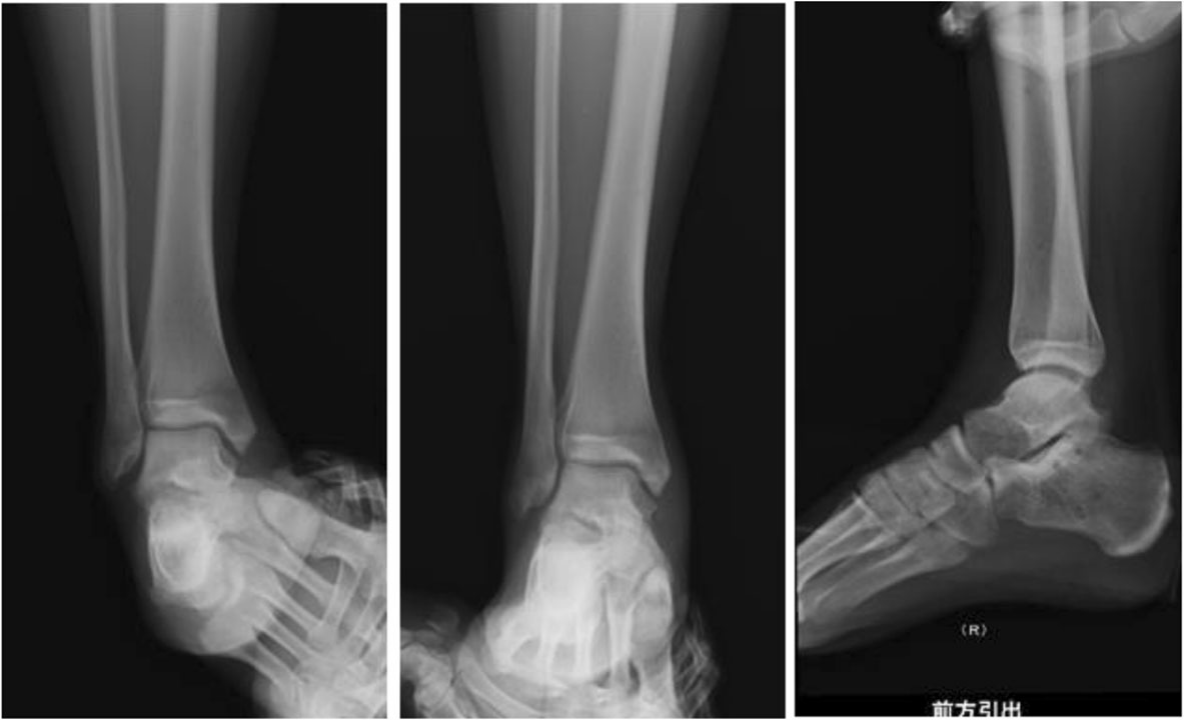
The varus and valgus stress views and anterior drawer view in X-ray 4 months postoperatively. Adequate stability of the ankle joint was observed

At 5 months postoperatively, the patient returned to basketball without any problems, and full range-of-motion was achieved with good ankle stability. The varus and valgus stress views and anterior drawer view on x-ray revealed adequate stability of the ankle joint (Fig. 4), and the JSSF ankle/hindfoot scale score was excellent (100/100).

## Discussion and conclusions

Pure ankle dislocations are extremely rare. In their systematic review, Wight et al. [1] reported the estimated incidence of pure ankle dislocation to be 0.065% (13/20,000) among presentations of ankle injury, with 73% (112/154) of the cases occurring in males.

Most pure ankle dislocations are closed dislocations [7] caused by high-energy trauma, such as motorcycle accidents, sports injuries, and falls from heights [8]. This case was an extremely rare open dislocation with 4.5-cm transverse wound at the distal fibula. The patient fell and injured her right ankle during contact play in basketball. We considered that the mechanism underlying the injury was axial loading by landing with plantarflexion and ankle inversion, similar to previous reports [9, 10]. Furthermore, because there were no fractures, it is possible that some traction force, joint and ligamentous laxity, and other factors may have been related to the mechanism. In fact, the patient had a history of one instance of sprained right ankle. Moreover, some reports [8] [7] have mentioned predisposing factors contributing to the pathogenesis of pure ankle dislocations, such as internal malleolus hypoplasia, ligamentous laxity, weak peroneal muscles, and repetitive ankle sprains.

Early reduction is important to achieve good clinical outcomes. Sayit et al. [11] reported using an external fixator for 6 weeks for reduction and treatment of ankle dislocation. The advantage of external fixation is the ease and safety of assembly at any time, even in an emergency situation such as in the present case. It also provides firm ankle joint fixation while improving swelling. In this case, we considered that external fixation was better than casting because severe ankle swelling and open wound were observed. There was a concern of neurovascular complication and infection after casting. An external fixation was only used for the first 2 weeks while the soft tissue and wound condition were observed. Although external fixation is an easy approach to achieve strong fixation, long-term use can be more uncomfortable for the patient compared with casting.

The necessity of repairing ruptured ligaments is disputable. In the study reported by Wight et al. [1], 46% of patients with ankle dislocation underwent nonsurgical treatment, whereas ligamentous repair was performed for 26% patients. Distefano et al. [12] and Uçar et al. [13] reported cases in which good clinical results were obtained with nonsurgical treatment. In contrast, Colville et al. [14] reported the case of a patient who presented with open dislocation and in whom the lateral ligaments were not repaired, with the outcome of moderate instability of the ankle and development of degenerative changes in the joint. In the present case, ligament repair with suture tape augmentation was performed because the patient was an elite athlete and wanted a swift recovery to return to playing basketball. Furthermore, both the ATFL and CFL were completely ruptured near the proximal attachment rather than just being partially torn.

The procedure of primary repair was performed with reference to the Broström technique [15]. After primary repair, suture tape augmentation was performed for both the lateral (ATFL and CFL) and medial sides (DL) of the ankle joint. This technique is a simple and quick procedure that can achieve adequate stability of the ankle joint. An effect similar to the internal brace can be expected by creating tibiocalcanear and tibiotalar anchor bridges with suture tape. This facilitates early range-of-motion training and prevents ankle contracture. In reference to strength of suture augmentation, Viens et al. [16] reported the ATFL with suture tape augmentation is at least as strong and stiff as the native ATFL at time zero. Furthermore, suture tape used in combination with the Broström repair provided increased strength and stiffness compared with the standard Broström repair, which produced an immediate strength of less than 50% of the intact ATFL in a cadaveric model.

The procedure of suture tape augmentation can also fine-tune the tension of the suture tape by finely adjusting the tape length when inserting the distal BioComposite SwiveLock® anchor. With the distal anchor is temporarily inserted, the tension of the suture tape can be checked and fine adjusted while moving the ankle joint under fluoroscopy. After that, the final insertion can be performed. Therefore, if the SwiveLock® anchors are inserted in the correct position of the anatomical attachment, safety and reliability of the procedure are high. There is no concern about postoperative ankle joint range-of-motion limitation by over-tensioning.

Another advantage of the system is that the SwiveLock®⎕ anchor is composed of polylactic acid and β-tricalcium phosphate; hence, management is straightforward even if further ligament reconstruction is required.

The previous systematic review [1] also reported a mean period of immobilization of just over 6 weeks, with the most common long-term adverse effect being decreased range-of-motion (which occurred in 18% of the cases). Rivera et al. [17] reported three cases of pure dislocation of ankle and observed a 10°–15° decrease in the range of dorsiflexion in two patients. These complications can reduce performance levels for high-level basketball players; thus, we aimed to prevent these complications in the present case. The total fixation time was therefore kept to a minimum. The present case demonstrates that early reduction, adequate ankle stability, and a short period of immobilization can prevent ankle contracture and result in good clinical outcomes, especially for athletes.

At 5 months postoperatively, the JSSF ankle/hindfoot scale score [5, 6] was excellent (100/100). Her position was guard in basketball and was required to dribble cut-in and jump shoot, but she was able to return to basketball without any problems. After an additional 2 months, she was able to participate and play in the Winter Cup 2019 (the national high school basketball tournament in Japan) at the previous performance level. Patient satisfaction was high because this tournament was her target and was to be the final tournament of her high school life.

At the final follow-up in our hospital, conducted at 9 months postoperatively, the patient exhibited excellent ankle function and maintained her performance level without degenerative arthritis. We report an excellent outcome of ankle dislocation in a young athlete that was treated using external fixation and ligament repair with suture tape augmentation. This approach can reduce the required period of external fixation and facilitate early return to high-performance sports.

## Data Availability

All data concerning the case are presented in the manuscript.

## Abbreviations

CT: Computerized tomography
ATFL: Anterior talofibular ligament
CFL: Calcaneofibular ligament
DL: Deltoid ligament
JSSF: Japanese Society for Surgery of the Foot
